# Supplementary material for: Antifungal and other bioactive properties of the volatilome of Streptomyces scabiei
Source: Appl Environ Microbiol. 2025 Oct 1;91(10):e00863-25. doi: 10.1128/aem.00863-25 (PMC12542781; doi:10.1128/aem.00863-25)
Supplement: Supplemental material — Figures S1 to S3; Table S1. [file aem.00863-25-s0001.docx]

**Supplemental Material**

**The antifungal and other bioactive properties of the volatilome of *Streptomyces scabiei***

Nudzejma Stulanovic^1^, Djulia Bensaada^2^, Loïc Belde^1^, Delphine Adam^1^, Marc Hanikenne^3^, Jean-François Focant^2^, Pierre-Hugues Stefanuto^2^, and Sébastien Rigali^1,*^

^1^InBioS – Center for Protein Engineering, Institut de Chimie, University of Liège, Liège B-4000, Belgium;

^2^Molecular System - Organic Biological Analytical Chemistry Group, University of Liège, Liège B-4000, Belgium;

^3^InBioS-PhytoSystems, Translational Plant Biology, University of Liège, B-4000 Liège, Belgium.

*Correspondence : [srigali@uliege.be](mailto:srigali@uliege.be)

**Supplementary Figure S1**

**
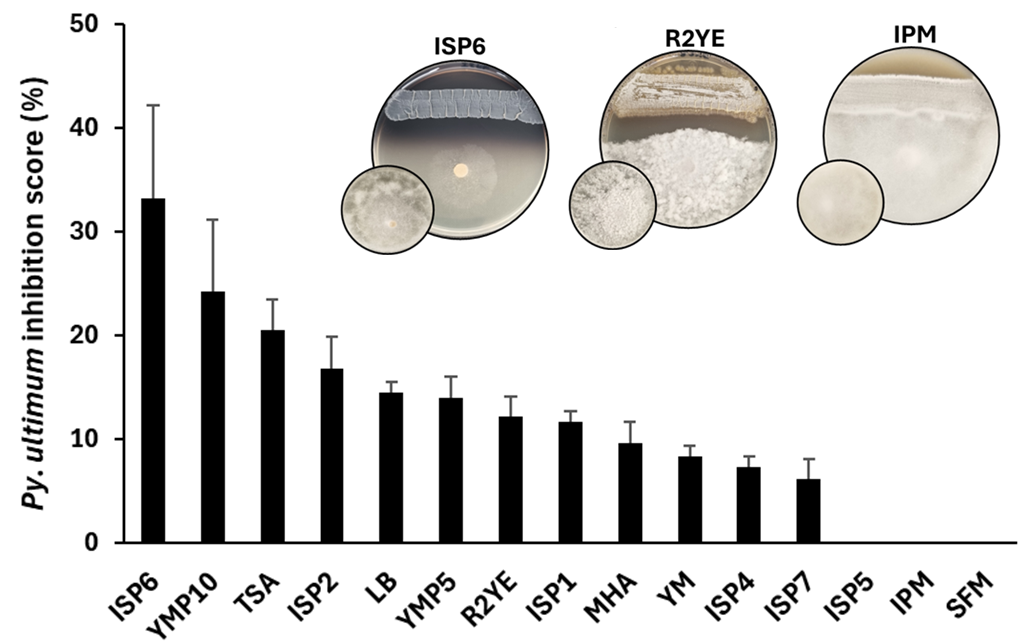
**

**Figure S1**: Semi-quantitative evaluation of antimicrobial activities of *S*. *scabiei* 87-22 against oomycete *Pythium ultimum* on 15 growth media. A score of 100 means full growth inhibition of the tested microorganisms, while a score of 0 indicates no growth inhibition. Insets in the bottom left corner are control plates which display the growth of tested microorganisms without *S. scabiei.*

**Supplementary Figure S2**


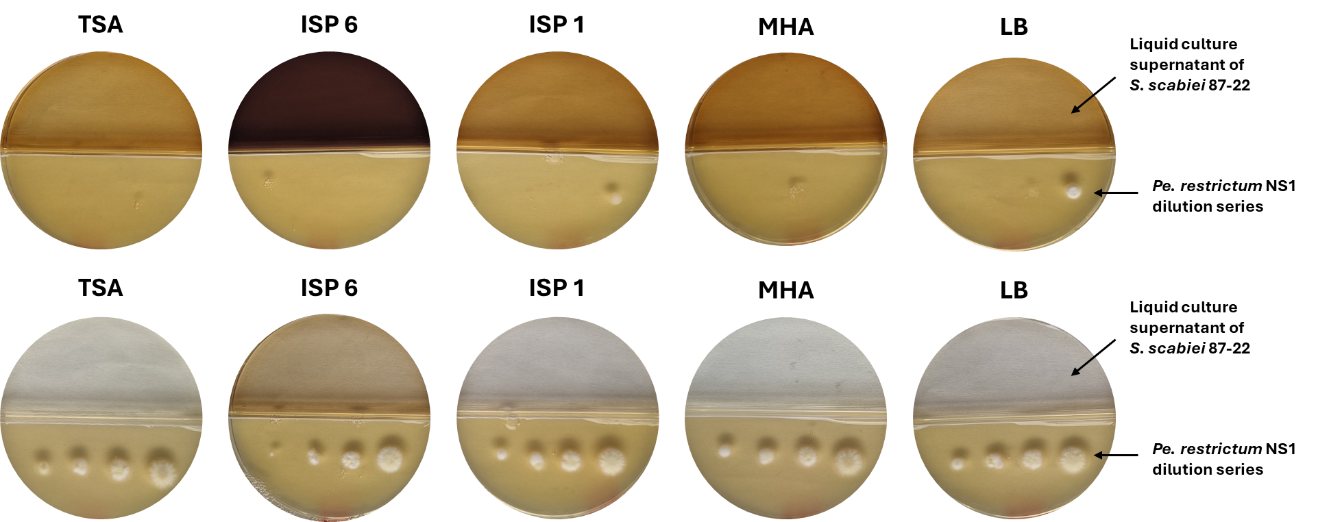


**Figure S2:** Evaluation of antifungal activity of VCs produced by *S. scabiei* in the five liquid culture supernatant using bicameral petri plates.

**Supplementary Figure S3**


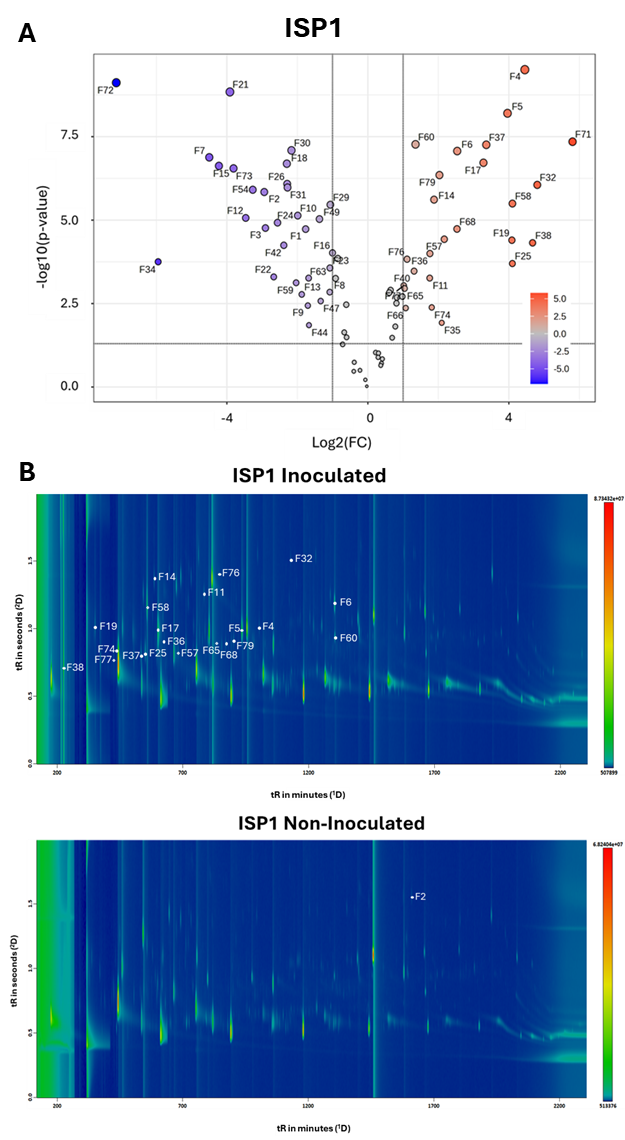


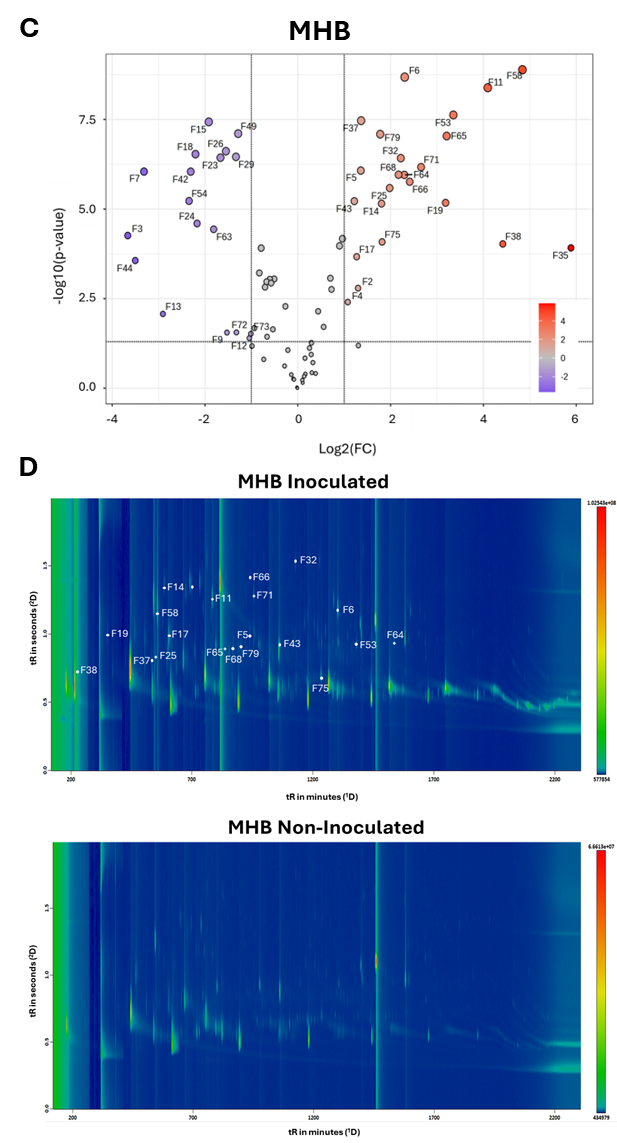


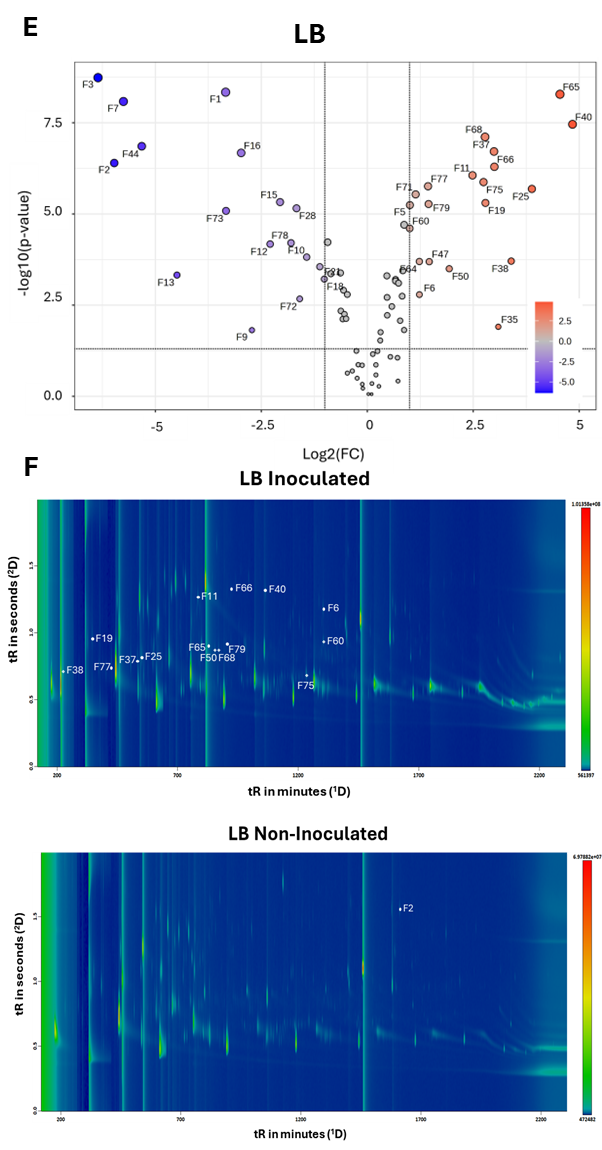


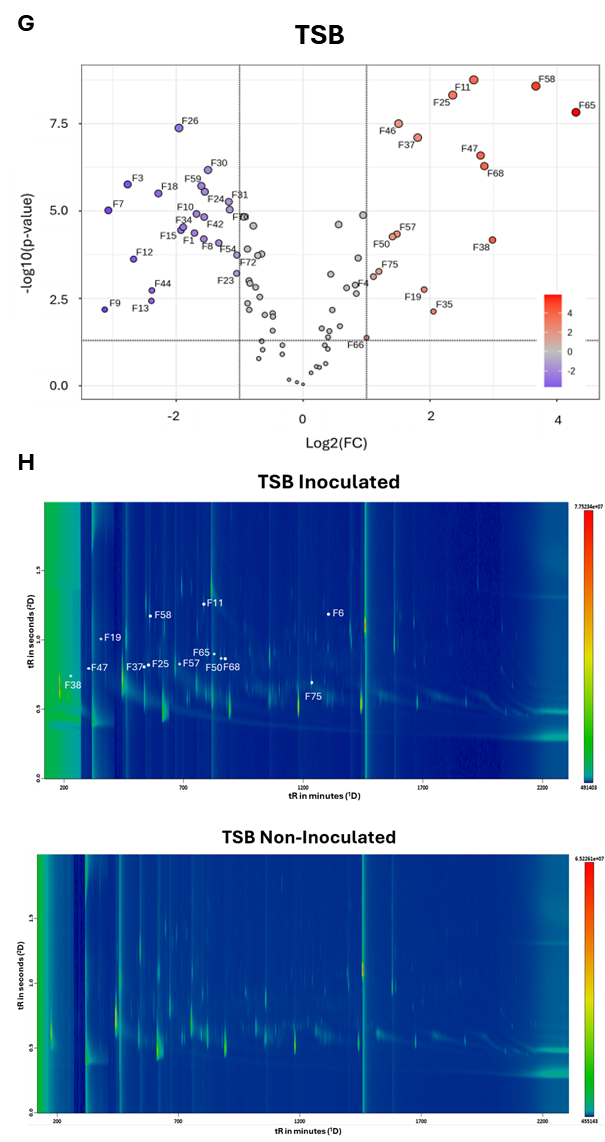


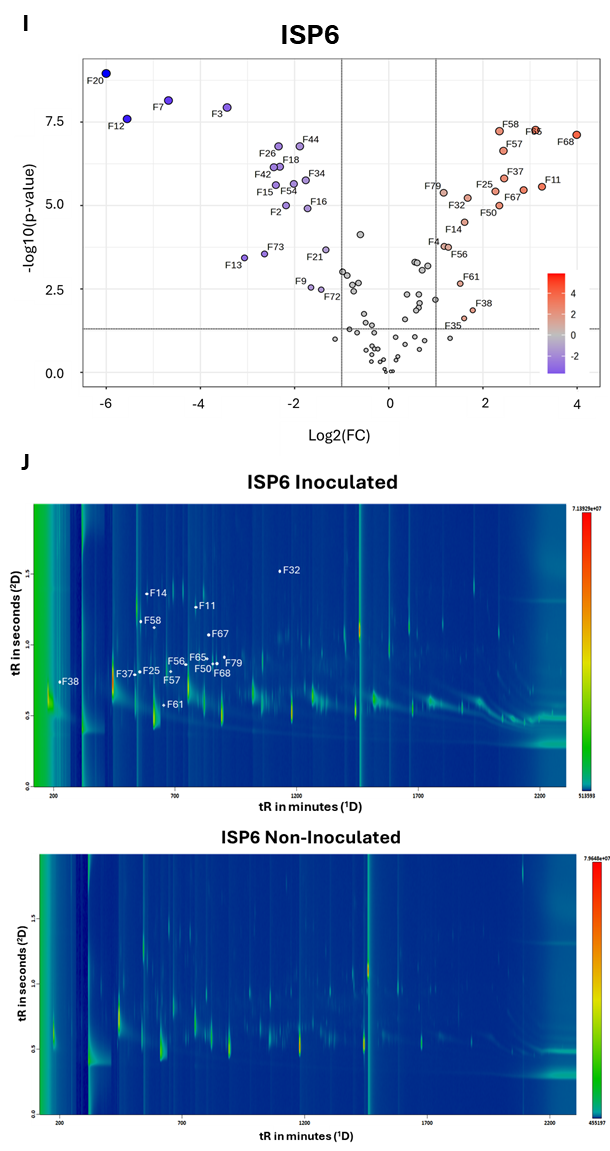


**Figure S3**. **Volcano plots and GC × GC -TOF MS chromatograms in ISP1 (A, B), in MHB (C, D), in LB (E, F), in TSB (G, H) and in ISP6 (I, J).** Upper panels: volcano plots of VCs identified by GC × GC-TOFMS across five media. The X-axis shows the Log₂ FC in VC intensity between medium non-inoculated (control) and inoculated by *S. scabiei* 87-22. The Y-axis displays the -log₁₀ (p-value), signifying the statistical significance of these changes (p-value ≤ 0.05 or -log_10_ p-value ≥ 1.3). The volcano plots are divided into three zones: 1) where the VCs do not result from the metabolic activity of *S. scabiei* 87-22 (Log_2_ FC < -1, blue circle); 2) where the log₂ FC did not significantly vary (-1 < Log_2_ FC < 1, grey circle), and 3) where result from the metabolic activity of *S. scabiei* 87-22 (Log_2_ FC ≥ 1, red circle). Middle and lower panels: chromatograms of inoculated and non-inoculated media are represented in lower panels. Each spot in the chromatogram corresponds to a VC separated by GC × GC: the first dimension (^1^D), using a non-polar column, separates compounds primarily according to their boiling points, while the second dimension (^2^D), using a mid-polar column, separates them based on their polarity. The area intensity of each compound is visualized using a color gradient, from light blue (lower intensities) to red (higher intensities), the dark blue color represents the background noise.

**Supplementary Table S1.
MSI-based identification levels and spectrometric data of volatile compounds with significant Log_2_ FC ≥ 1**

| **Feature number** | **Compound name** | **RI** | **RI Lib** | **Δ RI** | **Similarity** | **Reverse** | **MSI**^[[1]](#footnote-1)^ **Level** |
| --- | --- | --- | --- | --- | --- | --- | --- |
| F02 | 2-p-Tolylamino-cyclopent-1-enecarbonitrile | 1623.9 | / | / | 703 | 717 | 3 |
| F04 | Acetamide, N-(2,6-dimethylphenyl)- | 1222.9 | / | / | 785 | 802 | 3 |
| F05 | 2-Methylisoborneol | 1183.9 | 1197 | 13.1 | 724 | 728 | 3 |
| F06 | Geosmin | 1408.1 | 1431 | 22.9 | 838 | 880 | 3 |
| F11 | Benzoic acid, methyl ester | 1095.6 | 1096 | 1.6 | 940 | 942 | 2 |
| F14 | Benzonitrile | 985.1 | 985 | 0.1 | 967 | 967 | 2 |
| F17 | Pyridine, 2,4,6-trimethyl- | 993.1 | 991 | 2.1 | 959 | 962 | 2 |
| F19 | Methylthio-2-propanone | 850.6 | 863 | 12.4 | 879 | 879 | 2 |
| F25 | 2-Heptanone, 5-methyl- | 965.5 | 971 | 5.5 | 876 | 924 | 2 |
| F32 | Benzenecarbothioic acid, S-methyl ester | 1297.6 | 1299 | 1.4 | 939 | 939 | 2 |
| F35 | Dimethyl selenodisulfide | 1048.9 | 1047 | 1.9 | 705 | 750 | 3 |
| F36 | Benzenamine, N-(1-methylethyl) | 1007.8 | / | / | 741 | 741 | 3 |
| F37 | 2-Heptanone, 6-methyl- | 955.2 | 956 | 0.8 | 843 | 881 | 2 |
| F38 | 3-Penten-2-one | 777 | 735 | 42 | 930 | 943 | 1 |
| F40 | Acetic acid, 2-phenylethyl ester | 1259 | 1258 | 1 | 918 | 930 | 1 |
| F43 | 2-Undecanone | 1259 | 1294 | 35 | 848 | 854 | 3 |
| F46 | 2-Pentanone, 1-phenyl | 1312 | 1327 | 15 | 678 | 813 | 2 |
| F47 | 2-Hydroxy-3-pentanone | 821.8 | 821 | 0.8 | 826 | 882 | 2 |
| F50 | 4-Nonanone, 8-Methyl- | 1135.6 | / | / | 750 | 825 | 3 |
| F53 | 2-Tridecanone | 1460.8 | 1497 | 36.2 | 856 | 856 | 3 |
| F56 | 4-Octanone, 7-methyl- | 1073.3 | / | / | 826 | 866 | 3 |
| F57 | 4-Nonanone | 1038.9 | 1030 | 8.9 | 788 | 877 | 2 |
| F58 | Dimethyl trisulfide | 969 | 970 | 1 | 917 | 917 | 1 |
| F60 | Dodecanal | 1409.5 | 1409 | 0.5 | 916 | 930 | 2 |
| F61 | Decane, 4-methyl- | 1022.2 | 1060 | 37.8 | 857 | 876 | 3 |
| F64 | 2-Tetradecanone | 1569 | 1597 | 28 | 849 | 849 | 3 |
| F65 | 2,2-Dimethylheptane-3,5-dione | 1119.5 | / | / | 829 | 829 | 3 |
| F66 | Benzeneethanol, Beta-methyl- | 1174.7 | 1179 | 4.3 | 832 | 832 | 2 |
| F67 | Phenol, 4-(2-aminoethyl) | 1125.3 | / | / | 673 | 695 | 4 |
| F68 | 4-Decanone | 1144.8 | 1137 | 7.8 | 744 | 803 | 2 |
| F71 | Creosol | 1194.3 | 1193 | 1.3 | 912 | 912 | 1 |
| F74 | 3-Hexen-2-one, 5-methyl- | 900 | / | / | 887 | 913 | 3 |
| F75 | 2,5-Dimethylhexane-2,5-dihydroperoxide | 1364.6 | 1367 | 2.4 | 734 | 753 | 3 |
| F76 | Benzyl methyl ketone | 1129.9 | 1110 | 10.9 | 895 | 895 | 2 |
| F77 | 2-Heptanone | 893.1 | 891 | 2.1 | 924 | 941 | 1 |
| F79 | 2-Decanone | 1163.2 | 1193 | 29.8 | 784 | 803 | 3 |
| MSI: Based on The Metabolomics Standards Initiative (MSI), four identification levels were established, from Level 1 (highest confidence) to Level 4 (lowest), using criteria adapted for GC × GC data. These include the ^1^D ΔRT, which is the difference in first-dimension retention time between the reference standard and the compound from the sample; the RSI (Reverse Similarity Index), which compares the compound's mass spectrum to a library spectrum; and the ΔRI, the difference between the experimental and library retention indices.  Level 1: Identified Compounds  • Reliably identified using a commercial standard.  • Criteria: ΔRT (in-house) < 20 s, RSI > 800, and ΔRI < 20.  Level 2: Putatively Annotated Compounds  • No standard used.  • Identification based on spectral similarity with commercial libraries (MAINLIB, Replib, Nist_RI).  • Criteria: RSI > 800 and ΔRI < 20.  Level 3: Putatively Characterized Compound Classes  • Assigned to a known chemical class based on spectral similarity with compounds from that class.  Level 4: Low-confidence structural assignments  • Identified compounds based on spectral data with low RSI, reflecting low confidence in structural identification. | | | | | | | |

1. [↑](#footnote-ref-1)
